# Evaluation of SLE Susceptibility Genes in Malaysians

**DOI:** 10.1155/2014/305436

**Published:** 2014-02-18

**Authors:** Julio E. Molineros, Kek Heng Chua, Celi Sun, Lay Hoong Lian, Prasenjeet Motghare, Xana Kim-Howard, Swapan K. Nath

**Affiliations:** ^1^Arthritis and Clinical Immunology Research Program, Oklahoma Medical Research Foundation, 1025 NE 13th Street, Oklahoma City, OK 73104, USA; ^2^Department of Biomedical Science, Faculty of Medicine, University of Malaya, 50603 Kuala Lumpur, Malaysia; ^3^Department of Molecular Medicine, Faculty of Medicine, University of Malaya, 50603 Kuala Lumpur, Malaysia

## Abstract

Systemic Lupus Erythematosus (SLE) is a clinically heterogeneous autoimmune disease with strong genetic and environmental components. Our objective was to replicate 25 recently identified SLE susceptibility genes in two distinct populations (Chinese (CH) and Malays (MA)) from Malaysia. We genotyped 347 SLE cases and 356 controls (CH and MA) using the ImmunoChip array and performed an admixture corrected case-control association analysis. Associated genes were grouped into five immune-related pathways. While CH were largely homogenous, MA had three ancestry components (average 82.3% Asian, 14.5% European, and 3.2% African). Ancestry proportions were significantly different between cases and controls in MA. We identified 22 genes with at least one associated SNP (*P* < 0.05). The strongest signal was at HLA-DRA (*P*_Meta_ = 9.96 × 10^−9^; *P*_CH_ = 6.57 × 10^−8^, *P*_MA_ = 6.73 × 10^−3^); the strongest non-HLA signal occurred at *STAT4* (*P*_Meta_ = 1.67 × 10^−7^; *P*_CH_ = 2.88 × 10^−6^, *P*_MA_ = 2.99 × 10^−3^). Most of these genes were associated with B- and T-cell function and signaling pathways. Our exploratory study using high-density fine-mapping suggests that most of the established SLE genes are also associated in the major ethnicities of Malaysia. However, these novel SNPs showed stronger association in these Asian populations than with the SNPs reported in previous studies.

## 1. Introduction

Systemic Lupus Erythematosus (SLE) is a heterogeneous autoimmune disease, in terms of both clinical presentation and incidence and severity across ethnically diverse populations. Asians are among those with a greater risk of SLE and have more severe disease presentations such as lupus nephritis [1]. SLE has strong and complex genetic components. While several genomewide association studies (GWAS) have been reported for European SLE populations, few Asian GWAS have been performed [2–4]. Among European identified SLE loci were HLA loci *HLA-DRA* [5] and *ATG5* [5], immune signal transduction loci *BANK1* [6],* BLK* [5], *LYN* [5], TLR, and IFN pathway related loci *IFIH1* [7], *STAT4* [8], *TNFAIP3* [9], *IRF7* [5],* IRF8* [7], as well as *NCF2* [7], *IL10* [10], *PHRF1* [5], *CD44* [11], *ICAM1_ICAM4* [7], *TYK2* [7], and *UBE2L3* [5]. Loci identified through Asian GWAS include *ETS1* [12], *SLC15A4* [12], *IKZF1* [12], *RASGRP3* [12], *TNFSF4* [12], and *TNIP1* [10].

Malaysia has a population of around 28 million with three major ethnic groups (Malays (60.3%), Chinese (22.9%), and Indians (7.1%)). SLE patients and controls from Malaysia offer a unique opportunity to explore the effect of different ancestral backgrounds [13] on SLE genetic architecture. We explored association of SLE-associated loci identified through GWAS in two majority populations, Chinese and Malays. Given that these cohorts may be admixed, we expect that ancestry proportion may influence SLE association. Although previous studies [13–18] reported genetic associations with some candidate genes in Malaysians. To our knowledge, this is the first study which assessed SLE susceptibility genes using large scale targeted fine-mapping on Malaysian populations.

Our objective was to replicate and fine-map genetic association in 25 previously reported SLE susceptibility loci and to assess population structure and individuals admixture in the two ethnically distinct Malaysian cohorts.

## 2. Materials and Methods

### 2.1. Subjects and Genotyping

We genotyped 347 cases and 356 controls from the two major Malaysian ethnic groups (Malays (MA) and Chinese (CH)) using the Illumina custom designed ImmunoChip array [19] as part of a separate ongoing genetic association project. The ImmunoChip is a dense fine-mapping genotype array that contains ~196,000 SNPs from 184 genes associated with at least one of 12 autoimmune diseases, including SLE. Genotyping was conducted through the Genotyping Core Facility of the Oklahoma Medical Research Foundation (OMRF), Oklahoma City, USA. Subjects were recruited in compliance with the Internal Review Boards of OMRF and the University of Malaya Medical Centre. All SLE cases fulfilled the ACR criteria for SLE classification [20, 21]. Controls were matched by ethnicity and gender. Our CH cohort included 288 cases and 292 controls (187 males and 393 females); MA included 59 cases and 64 controls (48 males and 75 females) (Supplementary Table 1 in Supplementary Material available online at http://dx.doi.org/10.1155/2014/305436).

### 2.2. Quality Control

Individuals were removed from analysis if they were genetically related to other study subjects (*r* > 0.25), as estimated through the relatedness coefficient implemented in GCTA, or if they were outliers (mean ± 2 standard deviations) determined by principal component analysis. SNPs were excluded according to the following criteria: poor genotyping clustering, missing genotype rate greater than 90%, Hardy-Weinberg disequilibrium *P* < 0.001 in controls, or minor allele frequency below 0.5% (Supplementary Figure 1). SNP positions were aligned with HG19. The analysis set contained 6,991 SNPs from 25 previously reported genes genotyped on 580 CH and 123 MA unrelated individuals.

### 2.3. Population Structure

**Figure 1:**
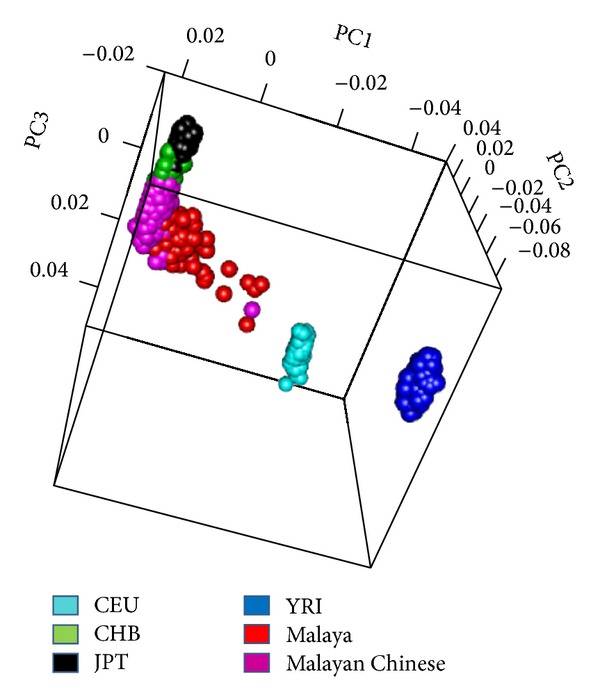
Principal components analysis of Chinese and Malay cohorts. Notably the Malaysian Chinese were a more homogeneous population than the Malays (CEU: North Europeans from CEPH; CHB: Beijing Chinese; JPT: Japanese; YRI: Yorubans from Nigeria).

In order to estimate population structure of our cohorts, we selected 14,134 SNPs with very low intermarker linkage disequilibrium (LD, *r* ^2^ < 0.2). This SNP set was enriched by variants with pairwise allele frequency difference >20%. We merged our cohorts with individuals from the 1000 Genomes Project (103 CEU, 100 CHB + 100 JPT, and 101 YRI). We estimated the first ten principal components using GCTA [22], as well as the mean and standard deviation for the first three principal components within each cohort (Figure 1). The same dataset was used to estimate individual admixture proportions in ADMIXTURE [23]. We estimated models of admixture using 1 to 7 ancestry components and determined the optimal admixture model by minimizing the cross-validation error using the Bayesian information criterion and the Akaike information criterion. Mean ancestry between cases and controls was compared with a two-tailed *t*-test.

### 2.4. Association Analysis

We performed individual SNP case-control association analysis using a chi-square statistic in PLINK [24]. Given the sample size of our cohorts and that this is a replication study, association was considered significant if *P* < 0.05 (alpha = 0.05). We guarded against type 1 error by performing permutation tests (100,000 permutations). Possible influence of admixture was corrected using a logistic regression model in PLINK [24] with the Asian ancestry proportion as a covariate. We used meta-analysis (Fisher's combined *P* value, four degrees of freedom) to combine association *P* values from both cohorts. For SNPs which were not significant in either cohort or when odds ratios were not in the same direction, no *P*_Meta_ was calculated. All associated SNPs passed the permutation test (results not shown).

The best SNP was selected for each region starting with the most significant combined *P*. We performed epistasis analysis using PLINK [24] and GAIA [25] in order to identify possible gene-gene interactions. We performed a conditional analysis using a logistic regression model (PLINK) for all significant SNPs from *STAT4* and *HLA-DRA* regions. We used the strongest associated SNP from each loci as the initial conditioned SNP to identify additional independent variants.

In order to check for additional sources of stratification, we used mixed models as implemented on EMMAX [26] (Supplementary Table 2).

RegulomeDB [27] and HaploReg [28] were used to identify functional elements overlapping with the selected SNPs.

### 2.5. Pathway Analysis

We chose to study five main pathways which contained the majority of the 25 target genes. All of these pathways are reported to be involved in SLE pathogenesis [29]. In order to determine if there were overrepresented pathways in these two cohorts we performed a gene set enrichment analysis (GSEA) weighted by the strength of the meta-analysis association using i-GSEA4GWAS [30]. The objective of this mode of GSEA was to identify the possible biological mechanisms that involve associated loci, and to identify candidate causal SNPs that affect the normal function in these pathways. Since we used a small set of loci we looked for pathways that contained at least two reported genes.

### 2.6. Power Analysis

We estimated the required sample size for detection of additional association signals in our cohorts using the method developed by Hoggart et al. [31] for alpha = 0.05. This method takes into account the effect of admixture on the probability of identifying an associated variant in a mixed population. The parameters included populations with similar characteristics as CH and MA (admixture proportions of 10% for CH and 20% for MA) and a power of detection of 80%.

## 3. Results

### 3.1. Population Structure

**Figure 2:**
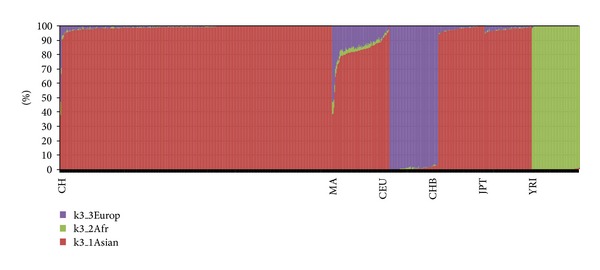
Admixture proportions of Chinese and Malays. Malaysian Chinese had less evidence of admixture from Europeans and Africans than Malaysian Malays (CH: Malaysian Chinese; MA: Malaysian Malays; CEU: North Europeans from CEPH; CHB: Beijing Chinese; JPT: Japanese; YRI: Yorubans from Nigeria).

Based on the proportions of the 1000 Genomes Project populations we estimated optimal population structure for Asian, African, and European ancestries. CH were very homogenous compared to the MA. As expected, mean Asian ancestral proportion was the highest in both populations (ASN_CH_ = 99.0 ± 3.2; ASN_MA_ = 82.3 ± 10.6), followed by European ancestry (EUR_CH_ = 0.7 ± 2.7; EUR_MA_ = 14.5 ± 9.4) and then African ancestry (AFR_CH_ = 0.3 ± 0.7; AFR_MA_ = 3.2 ± 1.6) (Figure 2). There was a significant mean ancestry difference between cases and controls in MA (case/control: 84.5/80.3: *P*_ASN_ = 0.02; 3/3.3: 12.5/16.4: *P*_AFR_ = 0.27; *P*_EUR_ = 0.02) but not in CH.

### 3.2. Association Analysis

We identified associated SNPs in 20 previously reported genes in either cohort. However, not all associated SNPs were significant in both cohorts. In CH published non-HLA loci SNPs showed significant association with SLE (Supplementary Table 3), including *ETS1* (rs1128334, *P*_CH_ = 2.4 × 10^−3^), *IRF8* (rs2280381, *P*_CH_ = 1.38 × 10^−2^),* TNFAIP3* (rs5029939, *P*_CH_ = 1.62 × 10^−2^), *STAT4* (rs3821236, *P*_CH_ = 1.86 × 10^−2^), and *RASGRP3* (rs13385731, *P*_CH_ = 3.63 × 10^−2^). In MA, *IKZF1* (rs4917014, *P*_MA_ = 1.06 × 10^−2^), *RASGRP3* (rs13385731, *P*_MA_ = 2.14 × 10^−2^), *KIAA1542* (rs4963128, *P*_MA_ = 2.25 × 10^−2^), *TNIP1* (rs10036748, *P*_MA_ = 2.55 × 10^−2^), and *IL21R* (rs3093301, *P*_MA_ = 3.28 × 10^−2^) were significantly associated with SLE. For the HLA locus, we replicated association for rs9271366 (*HLA-DRB1_HLA-DQA1* *P*_Meta_ = 1.33 × 10^−6^, *P*_CH_ = 2.62 × 10^−6^; *P*_MA_ = 2.92 × 10^−2^), consistent with the previous report on Malays and Chinese [13].

For all genes SNPs with the strongest association in this study differed from those previously reported. We identified 22 previously reported genes with at least one associated variant; the most significantly associated SNP for each gene was based on Fisher's combined *P* value. The strongest association was in the HLA region in the vicinity of *HLA-DRA* (rs6911777, *P*_Meta_ = 9.96 × 10^−9^, *P*_CH_ = 6.58 × 10^−8^, and *P*_MA_ = 6.73 × 10^−3^). The strongest non-HLA association was observed at *STAT4* (rs7568275, *P*_Meta_ = 1.68 × 10^−7^, *P*_CH_ = 2.88 × 10^−6^, and *P*_MA_ = 2.99 × 10^−3^). Also Asian identified *TNFSF4* (rs10798269, *P*_Meta_ = 5.98 × 10^−3^, *P*_CH_ = 3.87 × 10^−2^, and *P*_MA_ = 1.88 × 10^−2^) and *SLC15A4* (rs6486738, *P*_Meta_ = 4.88 × 10^−2^, *P*_CH_ = 0.122, and *P*_MA_ = 6.9 × 10^−2^) were replicated. We identified variants in LD with published variants *RASGRP3* (rs13425999, *r* ^2^ = 0.95, *P*_Meta_ = 6.82 × 10^−3^, *P*_CH_ = 3.30 × 10^−2^, and *P*_MA_ = 2.79 × 10^−2^), *TNIP1* (rs3792782, *r* ^2^ = 0.74, *P*_Meta_ = 2.09 × 10^−2^, *P*_CH_ = 0.181, and *P*_MA_ = 1.7 × 10^−2^), *C7orf72-IKZF1* (rs11185603, *r* ^2^ = 1, *P*_Meta_ = 2.3 × 10^−3^, *P*_CH_ = 7.61 × 10^−2^, and *P*_MA_ = 3.25 × 10^−3^). Even though these variants have a stronger association signal, they can be explained by their published counterparts.

We also identified a variant in *ETS1* (rs76404385, *P*_Meta_ = 2.05 × 10^−4^, *P*_CH_ = 3.41 × 10^−3^, and *P*_MA_ = 5.02 × 10^−3^) that was completely independent of published variant (rs1128334 *r* ^2^ = 0). European GWAS identified loci *IL10* (rs2232360, *P*_CH_ = 0.398, and *P*_MA_ = 5.06 × 10^−3^), *BANK1* (rs17031870, *P*_Meta_ = 1.26 × 10^−2^, *P*_CH_ = 2.89 × 10^−2^, and *P*_MA_ = 5.95 × 10^−2^), *PRDM1*-*ATG5* (rs9398065, *P*_Meta_ = 1.78 × 10^−3^, *P*_CH_ = 9.52 × 10^−3^, and *P*_MA_ = 1.95 × 10^−2^), *BLK-FAM167A* (rs11782375, *P*_Meta_ = 4.09 × 10^−4^, *P*_CH_ = 1.88 × 10^−4^, and *P*_MA_ = 0.194), *LYN* (rs7828258, *P*_Meta_ = 1.61 × 10^−2^, *P*_CH_ = 4.38 × 10^−1^, and *P*_MA_ = 5.18 × 10^−3^), *PDHX-CD44* (rs12362140, *P*_Meta_ = 7.27 × 10^−3^, *P*_CH_ = 7.46 × 10^−3^, and *P*_MA_ = 0.122), *ITGAM* (rs12444713, *P*_CH_ = 2.48 × 10^−3^, and *P*_MA_ = 0.71), *NCF2* (rs13306575, *P*_Meta_ = 1.17 × 10^−2^, *P*_CH_ = 8.11 × 10^−3^, and *P*_MA_ = 0.193), *IFIH1* (rs13023380, *P*_CH_ = 5.56 × 10^−2^, and *P*_MA_ = 1.42 × 10^−2^), *TNFAIP3* (rs5029928, *P*_Meta_ = 2 × 10^−2^, *P*_CH_ = 1.02 × 10^−2^, and *P*_MA_ = 0.287), *PHRF1* (rs4963128, *P*_CH_ = 0.4, and *P*_MA_ = 1.51 × 10^−2^), *IL21R* (rs8060368, *P*_CH_ = 3.59 × 10^−3^, *P*_MA_ = 0.172), *IRF8* (rs34912238, *P*_CH_ = 6.25 × 10^−3^, and *P*_MA_ = 0.115), and *ICAM1-ICAM4-TYK2* region (rs12975591, *P*_CH_ = 0.184, and *P*_MA_ = 3.06 × 10^−2^) also had a strong combined association with SLE (Table 1).

Notably, the scales of the odds ratio for rs7568275 (*STAT4*: OR_CH_ = 1.78, OR_MA_ = 2.36), rs9398065 (*PRDM1*-*ATG5*: OR_CH_ = 1.97, OR_MA_ = 3.31), rs5029928 (*TNFAIP3*: OR_CH_ = 1.88, OR_MA_ = 1.96), and rs76404385 (*ETS1*: OR_CH_ = 1.59, OR_MA_ = 3.25) were very close to *HLA-DRA* levels of OR (rs6911777 OR_CH_ = 2.26, OR_MA_ = 3.32).

Among the aforementioned 22 SNPs, we identified that rs11782375 (*FAM167A*_*BLK*) overlaps with an eQTL that potentially affects gene expression [32]. Additionally, rs13425999 (*RASGRP3*), rs5029928 (*TNFAIP3*), and rs11185603 (*IKZF1*) were identified as likely to affect binding by RegulomeDB [27]. These three SNPs contained enhancer and promoter histone marks in multiple cell types (in particular lymphoblastoid cell type GM12787) and also colocated with DNAse binding sites.

We used conditional analysis to identify multiple independent SNPs for each gene. In particular, *STAT4* had rs6740131 (*P*_CH_ = 2.89 × 10^−4^, *P*_CH_ = 5.76 × 10^−3^ after conditioning) as an additional independent SNP in CH. In the case of HLA, there were two additional independent SNPs in CH (rs2239806, *P*_CH_ = 9.3 × 10^−5^, and *P*_CH_ = 2.47 × 10^−6^ after conditioning and rs532098, *P*_CH_ = 7.44 × 10^−5^, and *P*_CH_ = 7.05 × 10^−5^ after conditioning).

### 3.3. Pathway Related Loci

We identified five important pathways involved in SLE pathogenesis which contained at least one of the 25 genes examined in our study. Both B- and T-cell function and signaling pathways had the greatest number of associated variants (Table 2). Neutrophil/monocyte function and signaling had four significantly associated SNPs, whereas TLR and type I IFN signaling pathways each included five genes with significantly associated SNPs. NF*κ*B signaling also contained SNPs significantly associated with SLE. We did not observe any significantly associated SNPs in DNA degradation apoptosis and clearance of cellular debris pathways.

The only significantly enriched pathway was hsa04514 [cell adhesion molecules (CAMs)]. We derived four causal SNPs that potentially explain enrichment of this pathway, where rs2071554 (nonsynonymous, coding (deleterious) in *HLA-DOB*) and rs1129740 (nonsynonymous, coding in *HLA-DQA1*) are candidate causal SNPs through their RECEPTOR_ACTIVITY/TRANSMEMBRANE_RECEPTOR_ACT-IVITY; rs8084 (essential splice site and intronic in *HLA-DQB*) and rs7192 (nonsynonymous, coding in *HLA-DRA*) were candidate causal SNPs through TRANSMEMBRA-NE_RECEPTOR_ACTIVITY.

### 3.4. Gene-Gene Interactions

We did not identify any gene-gene interaction between significant SNPs in either cohort.

### 3.5. Admixture Correction

We determined potential effects of admixture on associated variants within these pathways by adjusting case-control association analysis with admixture proportions. After admixture correction, only two MA SNPs were no longer significantly associated (*P* > 0.05). All CH SNPs passed the association threshold (*P* < 0.05).

## 4. Discussion

In this fine-mapping study we examined two understudied Malaysian populations to replicate previously known SLE genetic associations and to localize the most associated SNP within known SLE genes. Since SLE heterogeneity may be amplified in admixed populations, we adjusted association for admixture (Asian and European). We also categorized associated variants by pathways involvement and identified particular pathways with accumulation of reported associated variants in our Malaysian populations.

**Figure 3:**
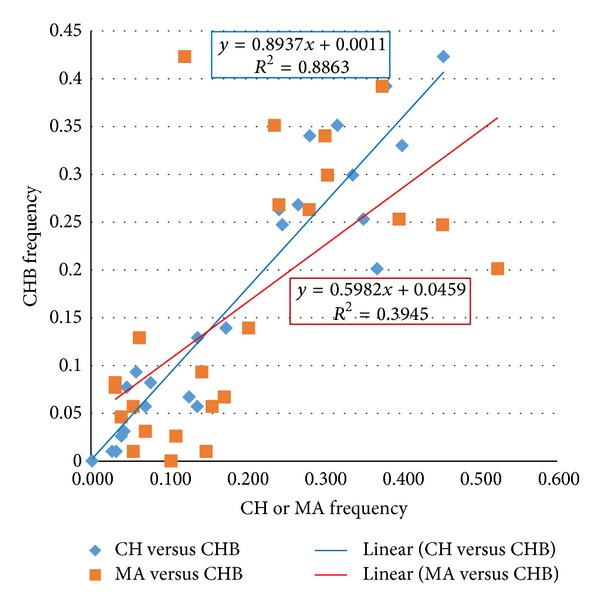
Comparison of allele frequencies of the top 25 SNPs from CH versus HapMap Chinese from Beijing (CHB) and MA versus CHB.

We found no effect of admixture on CH, which was not surprising since they are considered a homogenous population. In fact, the ancestry proportions of European and African were very small, and the minor allele frequencies for the top 25 genes were remarkably similar (Figure 3). Correlation between allele frequencies of CH versus CHB (*P* = 0.94) was higher than MA versus CHB (*P* = 0.65) further supporting our conclusion of the similarity between CH and CHB.

We replicated SLE association in *RASGP3* [12], *STAT4* [8], *TNIP1* [10], *IKZF1* [7], *IL21R* [33], *ETS1* [12], and *IRF8* [7]. It is not surprising that we did not identify more previously reported loci since the majority of loci were identified from studies of European and European American populations. Given the differences in LD structure between European and Asian populations, we identified new SNPs associated with SLE which could be either causal or in LD with the true causal SNPs within gene.

Associated variants were framed within their possible functional roles in immune-related pathways. The most important SLE-associated pathways in these populations were related to the B- and T-cell function and signaling pathways. We also introduced a causality model for gene set enrichment based on HLA SNPs in the cell adhesion molecules pathway (hsa04514). We identified four SNPs with potential functional effects through an eQTL and histone marks.

Although these results are encouraging, our study is limited due to the small sample size of our cohorts. Given the admixture proportions of these cohorts, we have estimated that we would require at least 1000 cases and controls to identify novel genomewide significant variants (*P* < 5 × 10^−8^) with moderate effects (OR > 1.5) for MA and almost double that for CH. Future large scale admixture mapping with the MA will be especially useful to identify novel SLE susceptibility genes. On the other hand, the CH population can be useful for straightforward association mapping for identifying novel genes or localizing the most likely causal variants.

In conclusion, our high-density fine-mapping on SLE targeted genes is one of the first such undertakings in Malaysian populations. Based on our rigorous analysis, we were able to replicate European and Asian SLE-associated loci in both Malaysian Malays and Malaysian Chinese and were able to identify additional variants that might serve as better tag SNPs for causal variants within these cohorts.

## Supplementary Material

Supplementary material contains three tables describing sample demographics; replicated SLE SNPs; and mixed model corrected association results for all our loci. Supplementary Figure 1 describes our approach to quality control through a flowchart.

## Figures and Tables

**Table 1:** SLE susceptibility genes in Malay (MA) and Chinese (CH).

Gene	Cytogenetic band	SNP	Base position	A1/A2	Chinese				Malay				Meta-analysis *P* value*
					F_A	F_U	*P* value	OR 95% CI	F_A	F_U	*P* value	OR 95% CI	
TNFSF4-LOC730070	1q25	rs10798269	173,309,713	A/G	0.342	0.401	3.87 × 10^−2^	0.78 (0.61–0.99)	0.314	0.460	1.88 × 10^−2^	0.54 (0.32–0.9)	5.98 × 10^−3^
NCF2	1q25	rs13306575	183,532,437	T/C	0.080	0.043	8.11 × 10^−3^	1.95 (1.18–3.22)	0.119	0.070	1.93 × 10^−1^	1.78 (0.74–4.28)	1.17 × 10^−2^
MAPKAPK2-IL10	1q31-32	rs2232360	207,040,659	A/G	0.328	0.351	3.98 × 10^−1^	0.9 (0.71–1.15)	0.576	0.397	5.06 × 10^−3^	2.07 (1.24–3.45)	—
RASGRP3	2p25.1-24.1	rs13425999	33,702,203	T/C	0.129	0.174	3.03 × 10^−2^	0.7 (0.51–0.97)	0.102	0.203	2.79 × 10^−2^	0.44 (0.21–0.93)	6.82 × 10^−3^
IFIH1	2q24	rs13023380	163,154,363	A/G	0.010	0.002	5.56 × 10^−2^	6.14 (0.74–51.13)	0.025	0.103	1.42 × 10^−2^	0.23 (0.06–0.82)	—
STAT4	2q32.2-32.3	rs7568275	191,966,452	G/C	0.453	0.317	2.88 × 10^−6^	1.78 (1.4–2.27)	0.422	0.236	2.99 × 10^−3^	2.36 (1.33–4.19)	1.68 × 10^−7^
BANK1	4q24	rs17031870	102,940,788	G/A	0.092	0.058	2.89 × 10^−2^	1.64 (1.05–2.56)	0.237	0.143	5.95 × 10^−2^	1.87 (0.97–3.59)	1.26 × 10^−2^
TNIP1	5q32-33.1	rs3792782	150,456,677	C/T	0.214	0.247	1.81 × 10^−1^	0.83 (0.63–1.09)	0.305	0.453	1.70 × 10^−2^	0.53 (0.31–0.89)	2.09 × 10^−2^
HLA-DRA	6p21.3	rs6911777	32,409,996	C/T	0.264	0.137	6.57 × 10^−8^	2.26 (1.67–3.05)	0.161	0.055	6.73 × 10^−3^	3.32 (1.34–8.21)	9.96 × 10^−9^
PRDM1-ATG5	6q21	rs9398065	106,546,034	C/G	0.075	0.039	9.52 × 10^−3^	1.97 (1.17–3.31)	0.119	0.039	1.95 × 10^−2^	3.31 (1.15–9.5)	1.78 × 10^−3^
TNFAIP3	6q23	rs5029928	138,189,942	T/C	0.083	0.046	1.02 × 10^−2^	1.88 (1.15–3.05)	0.059	0.031	2.87 × 10^−1^	1.96 (0.56–6.86)	2.00 × 10^−2^
C7orf721-IKZF1	7p13-11.1	rs11185603	50,306,810	G/C	0.236	0.282	7.61 × 10^−2^	0.79 (0.61–1.03)	0.144	0.302	3.25 × 10^−3^	0.39 (0.21–0.74)	2.30 × 10^−3^
BLK	8p23-22	rs11782375	11,294,934	C/T	0.277	0.380	1.88 × 10^−4^	0.62 (0.49–0.8)	0.297	0.375	1.94 × 10^−1^	0.7 (0.41–1.2)	4.09 × 10^−4^
LYN	8q13	rs7828258	56,867,945	T/C	0.223	0.242	4.38 × 10^−1^	0.9 (0.68–1.18)	0.136	0.281	5.18 × 10^−3^	0.4 (0.21–0.77)	1.61 × 10^−2^
KIAA1542-PHRF1	11p15.5	rs4963128	589,564	T/C	0.083	0.070	4.01 × 10^−1^	1.2 (0.78–1.86)	0.059	0.156	1.51 × 10^−2^	0.34 (0.14–0.84)	—
IRF7	11p15.5	rs7943546	612,148	C/T	0.024	0.033	3.99 × 10^−1^	0.74 (0.37–1.49)	0.025	0.055	2.46 × 10^−1^	0.45 (0.11–1.79)	3.26 × 10^−1^
PDHX-CD44	11p13	rs12362140	35,142,019	A/C	0.007	0.027	7.46 × 10^−3^	0.25 (0.08–0.75)	0.085	0.148	1.22 × 10^−1^	0.53 (0.24–1.2)	7.27 × 10^−3^
ETS1	11q23.3	rs76404385	128,333,055	T/C	0.202	0.138	3.41 × 10^−3^	1.59 (1.16–2.17)	0.178	0.063	5.02 × 10^−3^	3.25 (1.38–7.65)	2.05 × 10^−4^
SLC15A4	12q24.32	rs6486738	129,432,715	G/C	0.295	0.337	1.22 × 10^−1^	0.82 (0.64–1.05)	0.203	0.305	6.90 × 10^−2^	0.58 (0.32–1.05)	4.88 × 10^−2^
IL21R	16p11	rs8060368	27,412,414	T/C	0.012	0.039	3.59 × 10^−3^	0.3 (0.13–0.71)	0.170	0.109	1.72 × 10^−1^	1.66 (0.8–3.46)	—
ITGAM	16p11.2	rs12444713	31,378,235	A/G	0.284	0.369	2.48 × 10^−3^	0.68 (0.53–0.87)	0.5	0.52	7.10 × 10^−1^	1.1 (0.67–1.82)	—
ORF8-LOC100131952	16q24.1	rs34912238	86,001,903	T/C	0.039	0.077	6.25 × 10^−3^	0.49 (0.29–0.82)	0.076	0.031	1.15 × 10^−1^	2.56 (0.77–8.55)	—
ICAM1-ICAM4	19p13.2	rs5498	10,395,683	G/A	0.250	0.267	5.06 × 10^−1^	0.91 (0.7–1.19)	0.314	0.242	2.11 × 10^−1^	1.43 (0.82–2.51)	—
TYK2	19p13.2	rs12975591	10,627,814	G/A	0.415	0.454	1.84 × 10^−1^	0.85 (0.67–1.08)	0.232	0.121	2.62 × 10^−2^	2.19 (1.09–4.41)	—
UBE2L3	22q11.21	rs2236642	21,989,621	T/C	0.089	0.127	3.61 × 10^−2^	0.67 (0.46–0.98)	0.144	0.172	5.51 × 10^−1^	0.81 (0.41–1.62)	9.77 × 10^−2^

*When ORs from CH and MA are in different directions, we did not perform the meta-analysis.

— indicated SNPs where no meta-analysis was performed.

OR: odds ratio. Minor allele frequencies are given for the minor allele A1. F_A: minor allele frequency for cases; F_U: minor allele frequency for controls.

**Table 2:** Replicated genes and five immune-related pathways. Cells marked with an X represent presence of SNPs associated with SLE in those genes in either cohort. *marks genes present in the pathway.

Gene	B-cell function and signaling	Neutrophil and monocyte function and signaling	NF*κ*B signaling	T-cell function and signaling	TLR and type I IFN signaling
*ATG5 *			*		
*BANK1 *	X*				
*BLK *	X*				
*CD44 *				X*	
*ETS1 *	X*			X*	
*HLA-DR2 *	X*			X*	
*HLA-DR3 *	X*			X*	
*ICAMs *		X*			
*IFIH1 *					X*
*IKZF1 *	X*			X*	
*IL10 *	X*	X*		X*	
*IL21 *	X*			X*	
*IRF7 *					*
*IRF8 *	X*	X*			X*
*ITGAM *		X*			
*LYN *	X*				
*NCF2 *	X*				
*PHRF1 *					X*
*PRDM1 *	X*			X*	X*
*RASGRP3 *	X*				
*SLC15A4 *			X*		
*STAT4 *				X*	X*
*TNFAIP3 *			X*		
*TNFSF4 *				X*	
*TNIP1 *			X*		
*TYK2 *				*	
*UBE2L3 *			*		

Total	13	4	3	10	5
