# Supplementary material for: Evaluation of SLE Susceptibility Genes in Malaysians
Source: Autoimmune Dis. 2014 Feb 18;2014:305436. doi: 10.1155/2014/305436 (PMC3948475; doi:10.1155/2014/305436)

Supplementary Table 1. Sample Demographics

Gender	SLE Status	Chinese	Malaya
Male	Control	151	41
Female	Control	141	23
Male	Case	36	7
Female	Case	252	52
Male	Control	151	41
Female	Control	141	23
Male	Case	36	7
Female	Case	252	52

Supplementary Table 2. Replicated SLE associated loci. CH = Chinese; MA = Malaya; OR = Odds Ratio.

				Chinese		Malaya		Meta analysis
**Gene**	**CHR**	**Position HG19**	**Reported SNP**	**P value (288/292)**	**OR**	**P value (59/64)**	**OR**	**P value**
TNFSF4	1	173,191,475	rs2205960	1.44x10-1	1.20	8.84x10-1	1.05	3.89x10-1
NCF2	1	181799203	rs17849502	NA	NA	9.21x10-1	1.105	NA
IL10	1	206,939,904	rs3024505	9.70x10-1	1.01	6.68x10-1	1.31	9.29x10-1
RASGRP3	2	33,701,890	rs13385731	3.63x10-2	0.71	2.14x10-2	0.40	6.33x10-3
IFIH1	2	163,124,051	rs1990760	3.60x10-1	0.87	3.52x10-1	0.77	3.88x10-1
STAT4	2	191,902,758	rs3821236	1.86x10-2	1.32	1.16x10-1	1.55	1.54x10-2
BANK1	4	102,751,076	rs10516487	7.31x10-1	1.06	2.74x10-1	0.68	5.23x10-1
TNIP1	5	150,458,146	rs10036748	2.40x10-1	0.85	2.55x10-2	0.55	3.72x10-2
HLA-DRA	6	32,411,646	rs7192	6.29x10-1	1.07	1.08x10-1	0.67	2.50x10-1
PRDM1_ATG5	6	106,588,806	rs6568431	2.08x10-1	1.17	2.84x10-1	1.31	2.26x10-1
TNFAIP3	6	138,195,723	rs5029939	1.62x10-2	2.05	6.37x10-1	1.39	5.75x10-2
IKZF1	7	50,305,863	rs4917014	8.44x10-2	0.79	1.06x10-2	0.45	7.20x10-3
BLK	8	11,391,650	rs2248932	1.92x10-1	0.84	2.98x10-1	0.75	2.20x10-1
LYN	8	56,849,386	rs7829816	4.80x10-1	0.70	9.05x10-1	1.09	7.97x10-1
KIAA1542 (PHRF1)	11	589,564	rs4963128	4.05x10-1	1.20	2.25x10-2	0.35	5.19x10-2
IRF7	11	613,208	rs1131665	5.07x10-1	0.79	2.92x10-1	0.51	4.31x10-1
PDHX_CD44	11	35,084,592	rs2732552	8.70x10-1	1.02	3.19x10-1	0.69	6.34x10-1
ETS1	11	128,328,959	rs1128334	2.40x10-3	1.46	7.21x10-1	1.11	1.27x10-2
SLC15A4	12	129,300,694	rs1385374	7.63x10-1	1.05	1.57x10-1	1.69	3.73x10-1
IL21R	16	27,440,830	rs3093301	3.26x10-1	1.13	3.28x10-2	1.78	5.92x10-2
ITGAM	16	31,276,811	rs1143679	7.60x10-1	1.19	5.18x10-2	3.01	1.67x10-1
IRF8	16	86,018,633	rs2280381	1.38x10-2	0.61	1.21x10-1	1.83	1.23x10-2
ICAM1_ICAM4	19	10,397,403	rs3093030	4.31x10-1	0.90	5.78x10-1	1.17	5.95x10-1
TYK2	19	10,472,933	rs280519	9.95x10-1	1.00	2.29x10-1	0.73	5.65x10-1
UBE2L3	22	21,939,675	rs5754217	8.77x10-1	0.98	6.46x10-1	0.89	8.88x10-1

Supplementary Table 3. Mixed model corrected SNP association.

Gene	Cytogenetic Band	SNP	Base Position	Chinese P value	Malaya P value
TNFSF4 LOC730070	1q25	rs10798269	173,309,713	1.78E-01	3.53E-02
		rs2205960	173,191,475	4.53E-01	7.87E-01
NCF2	1q25	rs13306575	183,532,437	8.03E-03	1.54E-01
		rs17849502	181799203	-	9.01E-01
MAPKAPK2 IL10	1q31-32	rs2232360	207,040,659	4.44E-01	8.80E-03
		rs3024505	206,939,904	9.80E-01	9.58E-01
RASGRP3	2p25.1-24.1	rs13425999	33,702,203	4.20E-02	2.63E-02
		rs13385731	33,701,890	4.79E-02	2.63E-02
IFIH1	2q24	rs13023380	163,154,363	8.60E-02	4.23E-02
		rs1990760	163,124,051	3.75E-01	5.72E-01
STAT4	2q32.2-32.3	rs7568275	191,966,452	8.13E-05	5.51E-03
		rs3821236	191,902,758	1.45E-01	9.18E-02
BANK1	4q24	rs17031870	102,940,788	6.50E-02	2.69E-01
		rs10516487	102,751,076	9.71E-01	5.54E-01
TNIP1	5q32-33.1	rs3792782	150,456,677	1.86E-01	2.15E-02
		rs10036748	150,458,146	2.42E-01	2.71E-02
HLA-DRA	6p21.3	rs6911777	32,409,996	3.07E-04	1.03E-02
		rs7192	32,411,646	4.28E-01	2.79E-01
PRDM1 ATG5	6q21	rs9398065	106,546,034	1.40E-02	1.07E-02
		rs6568431	106,588,806	4.31E-01	2.62E-01
TNFAIP3	6q23	rs5029928	138,189,942	3.65E-02	4.18E-01
		rs5029939	138,195,723	4.85E-02	8.35E-01
C7orf72 IKZF1	7p13-11.1	rs11185603	50,306,810	1.20E-01	9.42E-03
		rs4917014	50,305,863	1.31E-01	1.27E-02
BLK	8p23-22	rs11782375	11,294,934	5.53E-03	2.64E-01
		rs2248932	11,391,650	6.24E-01	3.26E-01
LYN	8q13	rs7828258	56,867,945	5.21E-01	4.69E-03
		rs7829816	56,849,386	3.41E-01	8.78E-01
KIAA1542 (PHRF1)	11p15.5	rs4963128	589,564	3.15E-01	2.01E-02
		rs4963128	589,564	3.15E-01	2.01E-02
IRF7	11p15.5	rs7943546	612,148	4.63E-01	1.69E-01
		rs1131665	613,208	6.43E-01	1.90E-01
PDHX CD44	11p13	rs12362140	35,142,019	9.57E-03	1.98E-01
		rs2732552	35,084,592	5.79E-01	4.74E-01
ETS1	11q23.3	rs76404385	128,333,055	1.01E-02	1.53E-02
		rs1128334	128,328,959	2.82E-02	9.21E-01
SLC15A4	12q24.32	rs6486738	129,432,715	1.59E-01	5.60E-02

		rs1385374	129,300,694	6.24E-01	2.76E-01
IL21R	16p11	rs8060368	27,412,414	9.00E-03	7.40E-02
		rs3093301	27,440,830	5.09E-01	7.94E-02
ITGAM	16p11.2	rs12444713	31,378,235	1.26E-02	9.34E-01
		rs1143679	31,276,811	9.16E-01	5.14E-02
IRF8<br>*LOC100131952*	16q24.1	rs34912238	86,001,903	1.43E-02	7.17E-02
		rs2280381	86,018,633	2.70E-02	8.36E-02
ICAM1<br>*ICAM4*	19p13.2	rs5498	10,395,683	6.48E-01	3.61E-01
		rs3093030	10,397,403	6.84E-01	8.04E-01
TYK2	19p13.2	rs12975591	10,627,814	1.41E-01	2.21E-02
		rs280519	10,472,933	8.78E-01	2.75E-01
UBE2L3	22q11.21	rs2236642	21,989,621	6.31E-02	6.49E-01
		rs5754217	21,939,675	9.29E-01	4.35E-01

Supplementary Figure 1. Flowchart of quality control.

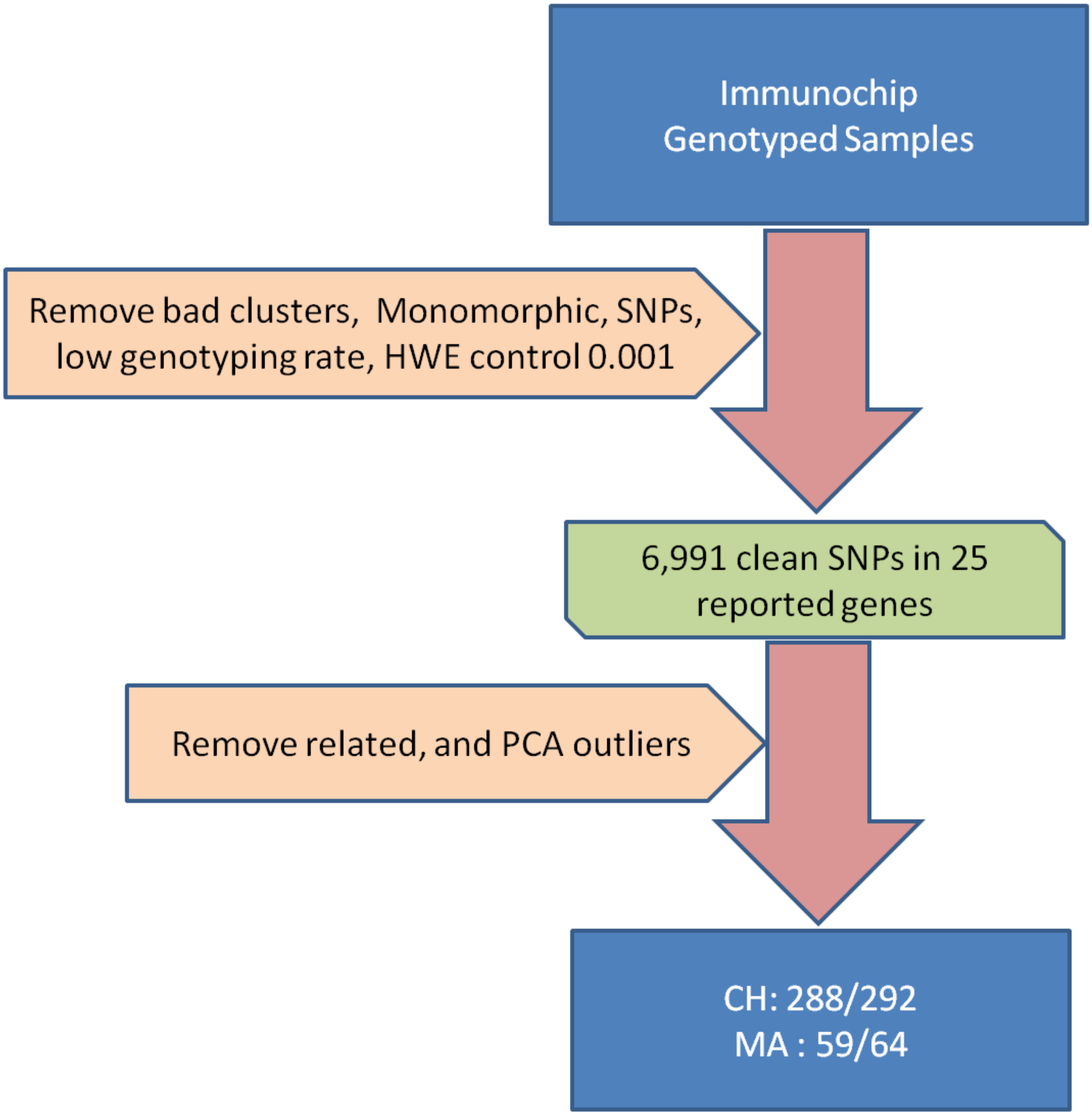

Supplement: Supplementary file 1 — Supplementary material contains three tables describing sample demographics; replicated SLE SNPs; and mixed model corrected association results for all our loci. Supplementary Figure 1 describes our approach to quality control through a flowchart. [file 305436.f1.pdf]
